# Development of Bread Dough by Sheeting: Effects of Sheeting Regime, Bran Level and Bran Particle Size

**DOI:** 10.3390/foods11152300

**Published:** 2022-08-02

**Authors:** Mohamed Otman Saleh Albasir, Mohammad Alyassin, Grant Murray Campbell

**Affiliations:** Department of Chemical Sciences, School of Applied Sciences, University of Huddersfield, Queensgate, Huddersfield HD1 3DH, UK; mohamed.albasir@hud.ac.uk (M.O.S.A.); m.alyassin2@hud.ac.uk (M.A.)

**Keywords:** dough development, bread quality, gluten, bran, sheeting

## Abstract

The effects of sheeting on bread dough development and baked loaf quality were investigated, using Dynamic Dough Density and springback to quantify development, and examining effects of the sheeting regime on bread quality in terms of loaf volume and crumb structure. Bread doughs, with and without bran at different levels and particle sizes, were formed through a short mixing period, then sheeted through a benchtop manual sheeter at roll gaps of 6, 9 and 12 mm for different numbers of sheeting passes. The sheeting of doughs without bran increased dough expansion and baked loaf volume up to 12 sheeting passes. Loaves were larger after sheeting at a 6 mm roll gap, reflecting the greater gluten development at the smaller gap, although the crumb structure was less fine, with fewer gas cells and larger average gas cell diameters. The addition of bran decreased dough expansion and loaf volumes, with Fine bran and Coarse bran both more damaging than Medium bran, indicating the opportunity to optimise bran particle size to maximise bread quality. Sheeting was effective in alleviating the damaging effects of bran, with sheeting for 8 passes giving more dough expansion, larger loaf volumes and finer crumb structures than sheeting for 12 passes, indicating an even more damaging effect of bran when gluten is overstretched by sheeting. The work demonstrates the opportunity to enhance bread quality, particularly of healthy high-fibre breads, by employing sheeting to enhance gluten development and to offset the damage to gluten caused by the presence of bran.

## 1. Introduction

Gluten quality is (along with wheat hardness) one of the great themes of cereal science [1,2]. Wheat, uniquely among cereals (with a small exception from rye), contains proteins that, when mixed with water, form a viscoelastic gluten network able to expand and retain gases during fermentation, allowing the dough piece to rise to create, on baking, a palatable aerated bread structure [2,3,4]. The unique ability of gluten to retain gases to give raised bread is the underpinning reason for the status of bread as the world’s most important food and wheat as the world’s most important cereal [5].

The gluten formed in wheat flour doughs can be developed by various means to enhance the ability of the dough to retain fermentation gases to give a large loaf with a fine crumb structure [2,3,4]. In traditional bulk fermentation breadmaking processes, the development of the gluten network occurs during the initial slow rising of the dough for several hours, which causes the gluten to be stretched and aligned. Following knock-back (or “punching”) to remove the gas and divide the bulk dough into individual pieces, the aligned gluten is now more effective at retaining gas during the final proving stage before baking, resulting in a larger loaf with a finer crumb structure.

In mechanical dough development (MDD) processes, high speed mixing in the presence of oxidants achieves dough development in the mixer within a few minutes, giving savings of time and higher bread yields, but at a significant energy cost [3,4].

The sheeting of doughs is mechanically more complex but energetically much more efficient, able to develop doughs with only a fraction of the energy required for high speed mechanical dough development while giving a fine and uniform crumb structure [6,7,8,9,10,11]. However, the practical challenges of implementing sheeting in industrial practice are such that commercial uptake has been limited [4], and dough development by sheeting has therefore been studied less extensively than MDD. 

Meanwhile, the inclusion of bran or other types of fibre in the dough formulation allows healthier breads, but compromises the aerated structure of the baked loaf by damaging gluten development, with the damaging effects strongly influenced by the bran particle size [4,12,13,14,15,16,17,18,19,20,21,22]. The superior gluten development that can be achieved by sheeting could potentially counteract the damage to gluten from fibre addition, to give high-fibre breads of greater consumer acceptability. The sheeting of bran-containing doughs has not been studied previously; hence, the current work aimed to investigate the effectiveness of sheeting to develop doughs containing the bran of different particle sizes, as a strategy for improving the quality of high-fibre and wholegrain breads.

## 2. Materials and Methods

The effects of sheeting and bran particle size and level on dough development and baked loaf quality were investigated. This section firstly describes the methods for preparing bran samples, doughs and baked loaves and for measuring dough expansion and baked loaf quality. It then describes the investigations undertaken using these methods.

### 2.1. Bran Milling and Particle Size Determination

Commercial Coarse wheat bran was obtained from Allinson Flour (Peterborough, UK). Following the practices of Zhang and Moore [16,17] and Campbell et al. [20,21] to obtain bran of identical composition but different particle sizes, the Coarse bran was milled to obtain Medium and Fine bran. A Retsch grinder ZM 1000 mill (Retsch UK Ltd., Hope Valley, UK) was used at a speed of 10,000 rpm and a screen aperture of 0.5 mm for milling the Coarse bran to obtain Fine bran, and a Newtry grain grinder (Newtry, UK) was used at a speed of 2600 rpm and load power 2000 W for 5 min for milling the Coarse bran to obtain a Medium bran.

The size distributions of the Coarse, Medium and Fine bran particles were measured in triplicate by sieve analysis using an Endecotts mechanical sieve shaker model EVS1 (Endecotts Ltd., London, UK) and stainless steel mesh sieves (2 mm, 1.7 mm, 1.4 mm, 710 μm, 355 μm, 180 μm, 90 μm and 53 μm). An amount of 100 g of each bran sample was placed on the top sieve and shaken for 15 min at a vibration intensity of 30%. The bran remaining on each sieve was collected and weighed to 0.1 g using an Ohaus balance.

### 2.2. Dough Preparation and Sheeting

Dough samples were prepared from white flour (100%, Allinson flour, Peterborough, UK), 1.5% sugar (ACROS, Thermo Fisher Scientific, Fair Lawn, NJ, USA), 4% yeast (Fast action dried yeast, Sainsbury’s, London, UK), 1.6% salt (Sainsbury’s cooking salt), 5% fat (Trex vegetable fat, Sainsbury’s), and 61% water (tap water, University of Huddersfield) for Control doughs. In bran-enriched doughs, wheat bran (Coarse, Medium or Fine) was substituted for white wheat flour at different percentages (5, 10 and 15%). The water absorption was increased by a percentage equal to half the percentage substitution of bran [19,20] for all particle sizes, as previous work had shown only a weak effect of wheat bran particle size on water absorption [16,19,20]. For example, for dough in which flour was substituted with 10% bran, the water absorption was increased by 5% to 66%, corresponding to 264 g of water for 400 g of (flour + bran).

Doughs based on 400 g flour were mixed prior to sheeting either in a low speed Henry Simon MajorPin mixer (Henry Simon Ltd., Stockport, UK) or in a high speed mixer called a Tweedy 1, a small-scale version of Tweedy mixers widely used in industry that use mechanical dough development to develop doughs [23]. Once formed to a sufficient degree of development, doughs were then further developed by passing them repeatedly through a sheeter. The MajorPin mixer would typically develop a dough over 15–20 min of mixing, the Tweedy 1 mixer typically over 3–4 min of mixing; the Tweedy 1 mixer imparts energy to the dough at roughly five times the rate of the MajorPin mixer.

**Figure 1:**
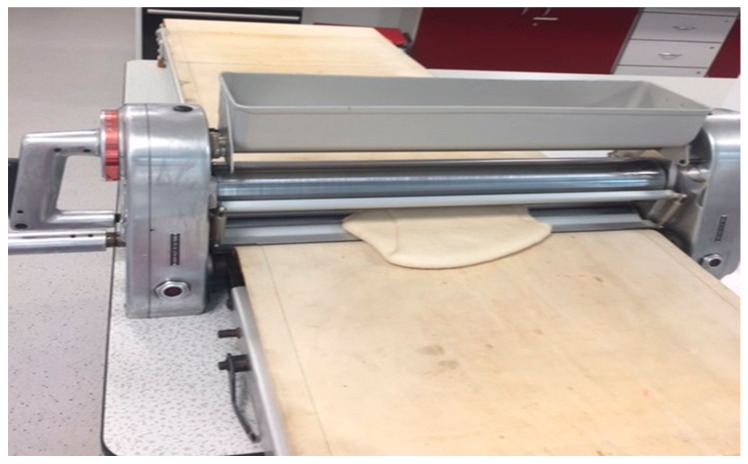
Rondo dough sheeter.

The Rondo table top sheeter (Rondo Bergdorf AG, Burgdorf, Switzerland), shown in Figure 1, is a hand-operated device that passes a dough piece between a pair of rolls from conveyor belts either side. The roll diameter is 37 mm, and the gap between the rolls can be varied from 23 mm down to 1 mm. Similar to [9], after each pass, the dough piece was folded, turned through a one-quarter turn, and passed back between the rolls (Kilborn and Tipples [6] had two sheeting passes at decreasing roll gaps before folding and turning). For Dynamic Dough Density sampling, a final pass (with folding, if necessary) at a gap of 12 mm was employed, to give a consistent thickness of dough for sampling.

### 2.3. Dynamic Dough Density Measurements

The ability of doughs to expand and retain gas was quantified by using the Dynamic Dough Density system (DDD). The DDD system measures the maximum expansion of a dough piece under conditions that mimic proving [19,20,24,25]. As the ability of dough to expand and retain gas arises from the development of the gluten, the DDD system offers a sensitive method for quantifying gluten development as affected by sheeting and bran addition.

After sheeting at the specified roll gap for a number of passes, samples for DDD testing were taken following the procedure of Campbell et al. [19,20]. The dough piece was gently sheeted to a final thickness of 12 mm, and four samples taken using a 21 mm diameter metal cookie cutter. Each sample was gently swirled in a spherical flask to strengthen the outer surface, weighed to 0.0001 g in the top cup of a double cup system, then transferred to the lower cup, immersed in xylene maintained at 38 °C in a jacketed beaker and weighed again. From the difference in weights, the sample’s density, *ρ*, is calculated as:

(1) $$\rho =\frac{m_{air}}{m_{air}-m_{xylene}}\rho_{xylene}$$

where *m_air_* and *m_xylene_* are the weights in air and immersed in xylene, respectively, and *ρ_xylene_* is the density of the xylene, which is 0.86 g cm^–3^ at 38 °C. Four DDD systems were run in parallel, and the changing weights as the dough samples expanded were recorded every 10 s on a computer running a LABVIEW 6.1 program. Recording commenced exactly five minutes after the end of mixing and continued for up to one hour. A plot of dough density versus time allowed the minimum density, corresponding to maximum expansion of the dough, to be determined.

### 2.4. Springback 

When bread doughs are passed through sheeting rolls at a set roll gap, the sheeted dough emerges thicker than the gap between the rolls, because the viscoelastic dough retracts after being stretched and squeezed through the rolls [10,26]. It was hypothesised that this “springback” (or “snap back” in Kempf et al.’s terminology [26], “recoil” in Qi et al.’s terminology [10]) might indicate the extent of gluten development, and that it might therefore be correlated with DDD expansion. If so, measurement of springback would be a more convenient way than DDD for quantifying the effect of sheeting on dough development.

In order to quantify springback, the thickness of the dough was measured by inserting a digital depth gauge (Model DDG100, Digital Micrometers Ltd., Sheffield, UK) into the sheeted dough. The thickness of the dough was averaged from four readings, and the springback calculated as:

(2) $$\mathrm{Springback}=\frac{\mathrm{Thickness}\;\left(\mathrm{mm}\right)}{\mathrm{Roll}\;\mathrm{gap}\;\left(\mathrm{mm}\right)}$$

### 2.5. Bread Baking

Doughs were prepared using the same recipes as for the DDD experiments. The doughs were mixed in the Tweedy mixer for three minutes, after which the doughs were sheeted through the Rondo sheeter at roll gap settings of 6, 9 and 12 mm for 4, 8 and 12 passes, along with a zero-pass sample obtained from the dough immediately from the mixer. From each sheet, four pieces were taken using a rectangular cutter to cut out dough pieces with dimensions of 6 cm × 12 cm. The samples were proved at 43 °C for 45 min, then baked at 175 °C for 27 min in a Hotpoint oven. The volume and texture of final baked loaves were measured by EinScan-SP 3D scanner and C-Cell Colour system, respectively.

### 2.6. EinScan-SP 3-D Scanner

The EinScan-SP 3D scanner (Shining 3D, Hangzhou, China) comprises a projector with a turntable onto which the sample is placed, connected to a computer that contains the software to collect and analyse the data. It was used in the current work to measure the volume of baked loaves using the AACCI standard method for volume measurements, based on three-dimensional imaging [27,28]. After taking images by the EinScan-SP 3D Scanner, the Meshmixer program (www.meshmixer.com, accessed on 1 February 2020) was used to calculate the final baked load volume. Baked loaves were weighed, and specific volume calculated as volume/weight (cm^3^/g).

### 2.7. C-Cell Image Analysis of Crumb Structure

The C-Cell Colour system (Calibre Control International Ltd., Warrington, UK) was used to quantify the crumb structure of the baked loaves. The C-Cell uses image analysis to quantify numerous elements of crumb structure (https://www.calibrecontrol.com/main-product-list/c-cell-colour, accessed on 1 July 2022). For the current study, it was expected that sheeting and the presence of bran particles would affect gluten development, and that this would show up primarily as effects on the number of cells, mean cell diameter and the mean cell wall thickness. After cutting the baked loaves into slices of 12 mm thickness using a bread slicer, the central three slices were used to conduct C-Cell crumb structure analysis. 

The above procedures and analysis systems were used to undertake three investigations: (i) effect of mixing and sheeting on dough expansion capacity; (ii) effects of bran particle size, level, sheeting roll gap and number of passes on dough expansion and springback; and (iii) effects of bran particle size, sheeting roll gap and number of sheeting passes on baked loaf quality. Fuller details and a wider range of experiments are reported in [29].

### 2.8. Effects of Mixing and Sheeting on Dough Expansion Capacity

Doughs were mixed for five minutes in the MajorPin mixer, to form a coherent dough with minimal gluten development, and sheeted for 3, 6, 9 and 12 passes at roll gaps of 6, 9 and 12 mm. After each sheeting pass, four samples were taken for DDD testing, along with zero pass samples from the dough taken immediately from the mixer. Runs were conducted in a random order, then repeated in the reverse random order, such that for each sheeting pass, eight samples were tested in the DDD system and the maximum expansion averaged.

Doughs were mixed in the Tweedy 1 mixer for 1, 2, 3 and 4 min, then sheeted at a roll gap of 6 mm for 1, 2 and 3 passes and, in a second experiment, for 2, 4 and 6 passes. As above, four samples were taken for DDD testing after each pass, along with zero pass samples from the dough taken immediately from the mixer, with runs undertaken in a random order and repeated in the reverse random order.

### 2.9. Effects of Bran Particle Size, Level, Sheeting Roll Gap and Number of Roll Passes on Dough Expansion and Springback

Doughs with different levels and particle sizes of bran were mixed in the Tweedy 1 mixer for 3 min, then sheeted at roll gaps of 6, 9 and 12 mm for 4, 8 and 12 passes. The total number of trials was (three bran sizes plus a Control) × (three levels) × (three roll gaps) × (three numbers of passes) = 108 trials, as shown in Table 1, with four samples taken for DDD testing from each trial.

The number of trials that can be completed in one day is limited by the relative slowness of the Dynamic Dough Density test, which typically takes 45 min. This experiment was therefore conducted over 9 days, with 12 trials per day, as shown in Table 1, blocked for bran level and roll gap, as it is well established that bran level and sheeting roll gap have large effects on dough development and bread quality, whereas the effects of bran particle size and number of sheeting passes are more subtle and less well known. The day-to-day variability of dough behaviour means that, strictly speaking, experiments from different days cannot be directly compared; however, the size of the differences from bran level and roll gap were expected to be sufficiently large relative to inter-day variability to allow broad comparisons to be made, while focussing on the more novel effects of bran particle size and number of sheeting passes. 

The nine days covered three different percentages of bran (5, 10 and 15%) and three roll gaps (6, 9 and 12 mm). Within each day, three particle sizes of bran (Coarse, Medium and Fine) were used in addition to the Control, and sheeted for 4, 8 or 12 passes.

Immediately after mixing, the doughs were sheeted through the Rondo sheeter at roll gaps of 6, 9 or 12 mm for 4, 8 or 12 passes. After each sheeting pass, the elongated dough piece was folded and turned before the next sheeting pass. (In principle this implies a constant reduction ratio of 2, but springback increased the dough thickness before folding such that the reduction ratio was greater than 2 and variable depending on the extent of springback.) After the final sheeting, when using the 6 and 9 mm gaps, the elongated dough piece was folded and passed through a 12 mm gap to get a consistent final thickness for DDD sampling while imparting minimal additional sheeting deformation (a folded 6 mm dough would be close to 12 mm for this final sheeting (slightly greater because of springback), such that sheeting to 12 mm imparts negligible additional deformation and development; a folded 9 mm dough becomes 18 mm plus springback, reduced relatively minimally to 12 mm). The 4, 8 and 12 total sheeting passes were undertaken in a random order, along with zero-pass samples from the dough immediately from the mixer. 

Springback was quantified by measuring the thickness of the dough after sheeting, and four replicate samples taken and their expansion measured in the DDD system.

### 2.10. Effects of Bran Particle Size, Sheeting Roll Gap and Number of Roll Passes on Baked Loaf Volume and Structure

The effects of bran and sheeting on bread quality (loaf volume and crumb structure) were investigated. Due to the greater complexity of baking trials and limited availability of the C-Cell (kindly lent by Calibre Control International), the baking trials did not investigate the full range of conditions of the above sheeting trials. Baking trials were performed using the same dough formulations, but just at a 10% level of Coarse, Medium and Fine bran addition, along with a Control sample without bran, and sheeted at just the two extreme roll gaps, 6 and 12 mm, each for 4, 8 and 12 passes. Thus, a total of 24 baking trials were performed (2 gaps × 3 passes × (3 bran particle sizes plus a Control)), with four loaves baked for each trial, as described above. The volume of each loaf and the crumb cell structure of three slices from the centre of each loaf were measured by the 3D scanner and C-Cell imaging, respectively. 

### 2.11. Statistical Analysis

Replicate DDD tests and springback measurements, either four or eight replicates depending on the investigation, were performed for each sample. For baking trials, four loaves were baked and their volumes measured, with three slices from each of the four loaves analysed by C-Cell. For each measurement, a pooled standard deviation was calculated, and error bars presented as ±1 standard deviation of the mean. 

## 3. Results and Discussion

The Dynamic Dough Density test measures the changing density of a yeasted dough sample as it expands under conditions mimicking proving; the minimum density indicates the maximum ability of the dough to expand [19,20,21,23]. In the current work it was employed to give an indication of the degree of gluten development as affected by sheeting and bran addition.

### 3.1. Effects of Mixing and Sheeting on Dough Expansion Capacity

**Figure 2:**
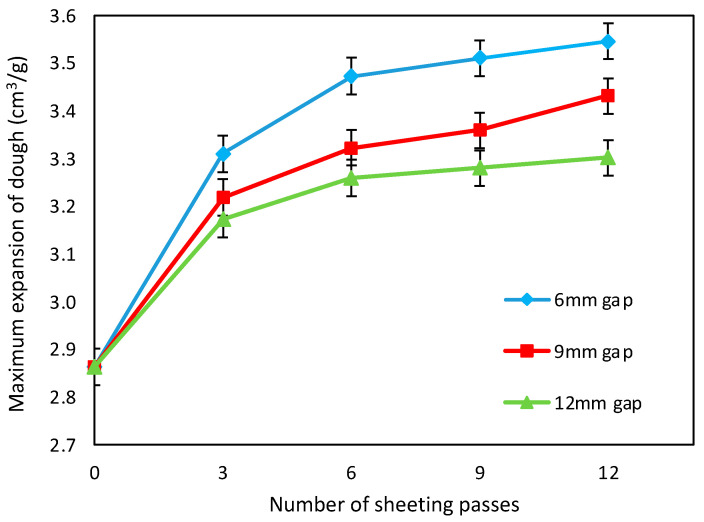
Maximum expansion against number of sheeting passes at a roll gaps of 6, 9 and 12 mm, following initial dough formation in the MajorPin mixer.

Figure 2 shows the maximum dough expansion (inverse of the minimum DDD density) against number of sheeting passes (3, 6, 9 and 12 passes) at different roll gaps (6, 9 and 12 mm) for doughs initially prepared in the MajorPin mixer (corresponding to zero sheeting passes). Clearly, sheeting the dough dramatically enhanced its ability to expand and retain gas, with increased gluten development following each additional pass. Sheeting for 12 passes at a roll gap of 12 mm increased the expansion capacity of the dough by 15.3%, compared with the undeveloped dough, and at roll gaps of 9 and 6 mm, the increases were 19.8% and 23.8%, respectively, showing more effective gluten development at the smaller roll gaps.

Kilborn and Tipples [6] sheeted for up to 82 passes, and Morgenstern et al. [9] for up to 40 passes for their weak flour dough and 100 passes for their strong flour dough, more than in the current work. Kilborn and Tipples [6] showed that the high efficiency of gluten development by sheeting can eventually lead to overmixing and gluten breakdown. In the current work, sheeting continued to be effective in enhancing dough expansion capacity up to 12 passes; more sheeting passes would be expected eventually to show a reduction in DDD expansion.

The smallest roll gap, 6 mm, gave the most effective gluten development. It might be thought that this is because it gave the most severe deformation of the dough. However, the extent of deformation depends on the reduction ratio, the ratio of the dough thickness entering the sheeter and the roll gap. Because the doughs were folded between sheeting passes, the reduction ratio was, to a first approximation, relatively constant at 2, and the deformation relatively constant. Springback was not measured in these trials, but in the studies described later, springback for the Control doughs without bran was 1.98 at the 6 mm roll gap, 1.86 at the 9 mm roll gap and 2.75 at the 12 mm roll gap after 12 passes. Therefore, the reduction ratio, after folding the doughs, was around 3.8–4 for the smaller roll gaps and 5.5 at the 12 mm roll gap, such that deformation was more severe at the larger gap. Therefore, the severity of the deformation is not the explanation for the greater development at the smaller roll gaps.

Another factor is the varying ratio of gap to roll diameter, which in principle gives a different deformation profile that could affect gluten development and springback. Engmann et al. [30] concluded that sheeted thickness relative to roll gap (in other words, springback) is much less sensitive to roll diameter/roll gap ratio than to reduction ratio. In the current work, the roll diameter was 37 mm, smaller than the 100 mm used by Engmann et al. [30] and the 85 mm used by Morgenstern et al. [9], so the ratio of roll diameter/roll gap may have had a greater effect.

Levine [31] showed that the gas content of the dough affects the degree of springback. In the current work, dough density increased from about 1.164 g cm^–3^ to 1.172 g cm^–3^ from zero to eight sheeting passes, then began to decrease (data not shown, but available in [29]), indicating a small degree of degassing during sheeting. The density change corresponds to a change in gas content of from about 7.6% to 7.0%, insufficient to have had a significant effect on the observed springback.

The Tweedy 1 mixer employs mechanical dough development in which rapid work input develops the gluten, typically in about 3 min of mixing. The development of gluten via high speed mixing is a more efficient alternative to traditional bulk fermentation, in which gluten is developed through slow stretching of the dough during the initial long fermentation stage, following by knock-back/punching and a shorter second proof. However, it was of interest in the current work to determine whether the high degree of dough development achieved by the Tweedy 1 mixer could be enhanced by subsequent sheeting.

**Figure 3:**
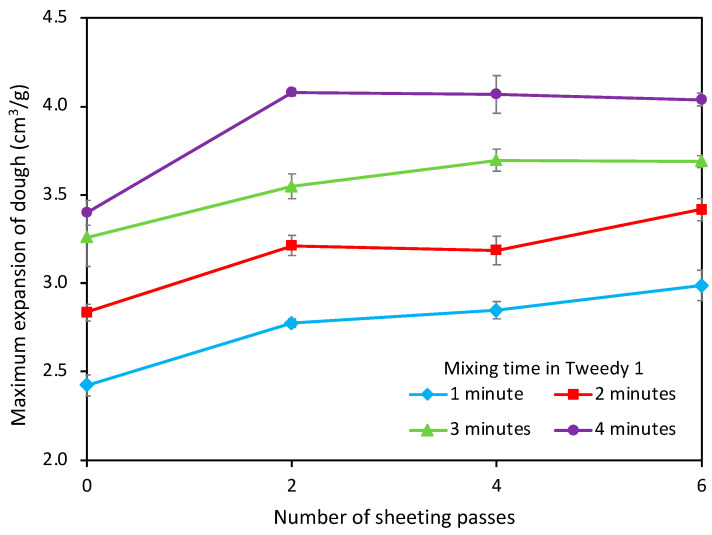
Maximum expansion against number of sheeting passes at a roll gap of 6 mm, following initial dough formation for 1, 2, 3 or 4 min in the Tweedy mixer.

Figure 3 shows the DDD maximum expansion of the dough after mixing in the Tweedy mixer for 1, 2, 3 or 4 min, followed by sheeting through a 6 mm roll gap. The trials were done in two ways—sheeting for 1, 2 and 3 passes, and sheeting for 2, 4 and 6 passes. The two trials were done on different days and, as is the nature with dough studies, are not directly comparable, but they confirm the same trends: that mixing for longer in the Tweedy 1 leads to greater expansion capacity, and that sheeting following mixing is able to increase the expansion capacity further. After 4 min of mixing in the Tweedy mixer and 3 sheeting passes, the limit of the expansion capacity appears to have been reached; further sheeting gave no further increase expansion capacity. However, the sheeting of doughs mixed for shorter times was unable to achieve the level of gluten development achieved by mixing for 4 minutes and then sheeting; it appears that, despite its efficiency, sheeting on its own cannot achieve as much development as high speed mixing followed by sheeting.

A combination of mechanical dough development followed by sheeting offers the potential for maximum gluten development and enhanced bread quality, although the improved bread quality is unlikely to be sufficient to justify the additional cost and complexity of implementing sheeting into the process. In the case of wholemeal and high-fibre breads, however, where bran damages bread quality and consumer acceptability, the opportunity to use sheeting to restore bread quality through enhanced gluten development may be commercially attractive.

### 3.2. Effects of Bran Particle Size, Level, Sheeting Roll Gap and Number of Roll Passes on Dough Expansion and Springback

The median particle sizes of the Coarse, Medium and Fine brans were determined from sieve analysis as 1262 μm, 385 μm and 174 μm, respectively, comparable with the 1182 μm, 585 μm and 210 μm used by Campbell et al. [21] in similar work, and covering a wider range than the 609, 415 μm and 278 μm used by Zhang and Moore [17]. The three brans were added to dough formulations at levels of 5, 10 and 15% of flour weight, and their effects on gluten development and baked loaf quality investigated compared with Control doughs.

**Figure 4:**
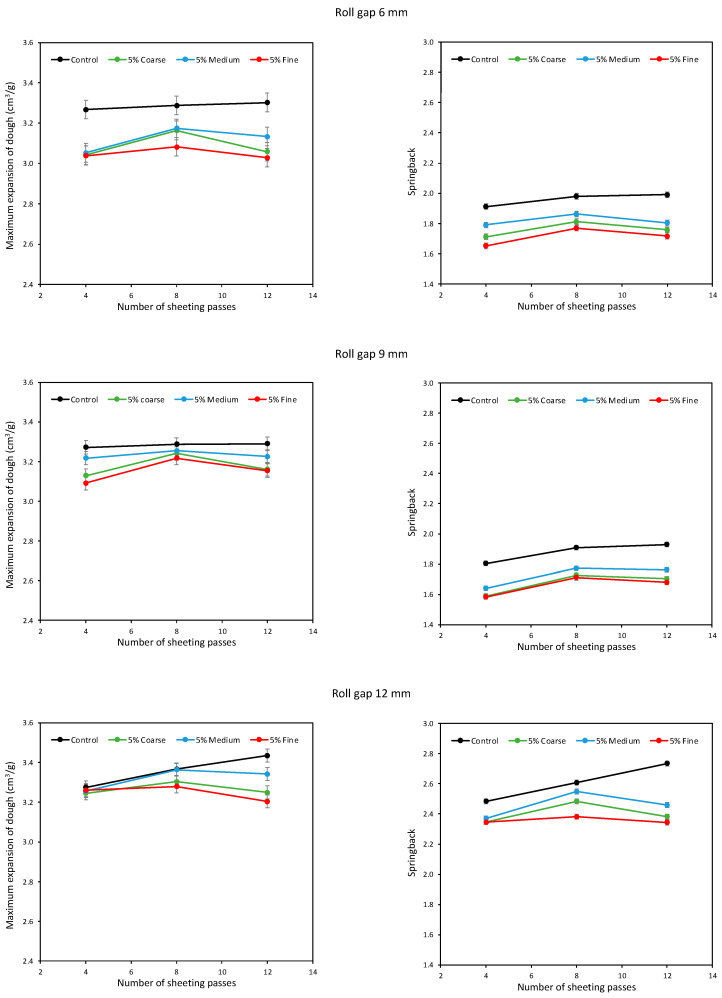
Average expansion (**left**) and springback (**right**) of doughs containing 5% Fine, Medium and Coarse bran, sheeted at roll gaps of 6 (**top**), 9 (**middle**) and 12 mm (**bottom**).

Figure 4 shows the maximum DDD expansion and springback at the 5% level of bran addition. Considering the Control dough with no bran, it is clear that sheeting increased both the expansion and springback of the dough. The addition of bran decreased expansion and springback, in line with previous literature reports [19,20,21]. Fine bran was consistently the most damaging to expansion and springback, while Medium bran was consistently the least damaging, with Coarse in between. This is in contrast to Campbell et al. [21], who found Coarse bran gave the most expansion and the largest baked loaves; however, that study was for doughs developed by mixing, where it was speculated that the greater aeration of the dough with Coarse bran particles enhanced gluten oxidation. In both studies, the results imply that it is possible to minimise the damage to dough development caused by the presence of bran particles by optimising the size of the bran particles, in agreement with Zhang and Moore [16,17]. It seems that, for sheeted doughs, Coarse bran particles are damaging because of their large size, while Fine bran particles are damaging because of their large number and that it is possible to identify an intermediate particle size that minimises the damage.

This effect of bran particle size was generally consistent across all three roll gaps and all three numbers of sheeting passes. However, in contrast to the Control dough, the effect of sheeting the doughs with bran was initially to increase expansion from 4 to 8 passes, but then to decrease expansion on prolonged sheeting to 12 passes. So, sheeting is effective at developing gluten, as established by previous workers [6,9,11,32,33], but the presence of bran disrupts the ever more stretched gluten sheets if the sheeting is prolonged. The efficiency of sheeting can go some way to enhancing gluten development, but in the presence of bran particles there is a limit beyond which further sheeting starts to be counter-productive.

Comparing the top (6 mm roll gap), middle (9 mm) and bottom (12 mm) graphs in Figure 4, there is not much difference in DDD expansion between the three roll gaps. Interestingly, at 12 mm and 4 sheeting passes, the DDD expansion of all four doughs (Control and with the three different particle sizes) was similar, only diverging after 8 and 12 passes. At the 5% level of bran addition, the doughs are not greatly different from the Control dough, hence similar expansions after 4 sheeting passes at 12 mm, but further sheeting starts to allow the interactions between the bran particles and the developing gluten network, and the influence of particle size on this interaction, to become apparent.

The springback results show a similar pattern between the different bran particle sizes at 12 mm and 4 sheeting passes, although in this case the Control dough gave more springback than the doughs with 5% bran. In general, sheeting at 12 mm gave greater springback than at 6 mm, with 9 mm appearing to give the lowest springback, although this trend is not reflected in the DDD expansion data; the 12 mm roll gap gave much larger springback values, but only slightly larger DDD expansion. However, it is emphasised that these experiments were done on different days and can only be compared with cautious awareness of the inherent day-to-day variability of dough studies. The springback values are around 1.8–2 for the Control dough at 6 and 9 mm roll gaps, increasing to as high as 2.75 for the 12 mm roll gap after 12 sheeting passes, in line with typical values reported by Kempf et al. [26].

**Figure 5:**
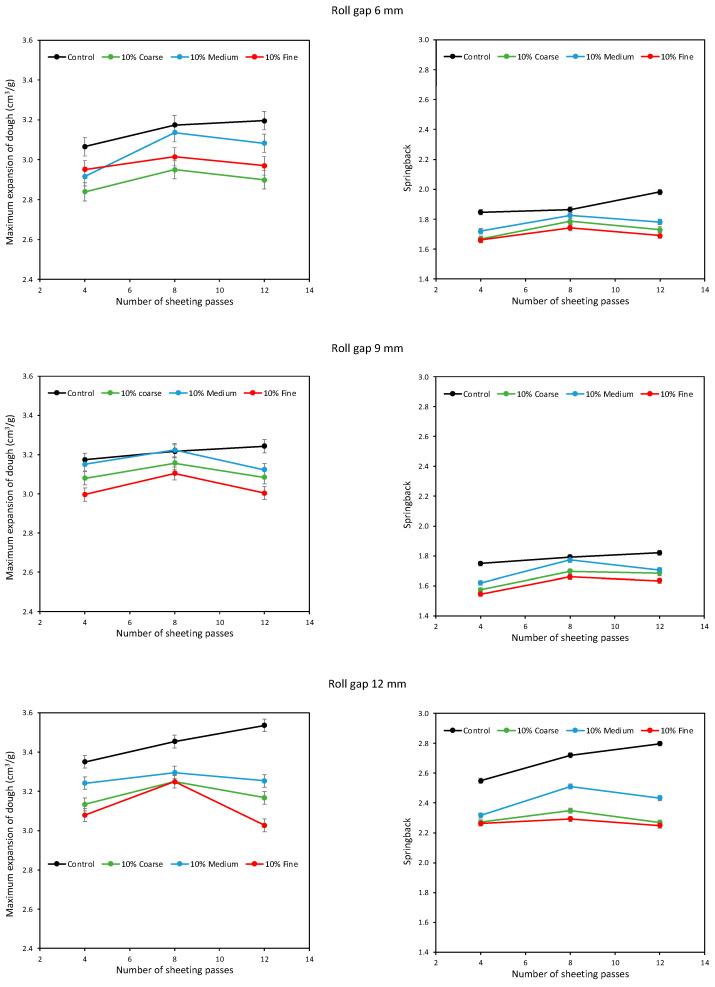
Average expansion (**left**) and springback (**right**) of doughs containing 10% Fine, Medium and Coarse bran, sheeted at roll gaps of 6 (**top**), 9 (**middle**) and 12 mm (**bottom**).

**Figure 6:**
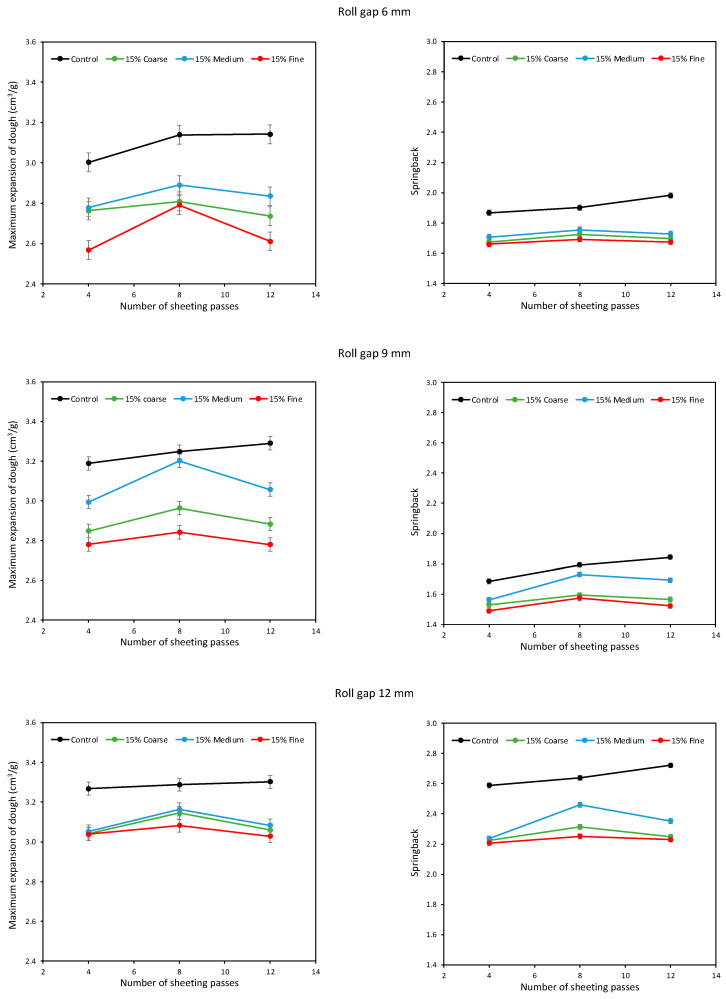
Average expansion (**left**) and springback (**right**) of doughs containing 15% Fine, Medium and Coarse bran, sheeted at roll gaps of 6 (**top**), 9 (**middle**) and 12 mm (**bottom**).

In general, the results at the higher levels of bran addition show the same patterns, magnified because of the higher bran levels. In Figure 5 and Figure 6, again the Fine bran reduced expansion and springback the most, and the Medium bran the least. Again, it is evident that sheeting for 8 passes gave greater expansion and springback than after only 4 passes, for all roll gaps, but that further sheeting to 12 passes decreased expansion and springback. The consistency of these patterns across all the conditions gives confidence in the conclusion that there is an intermediate particle size and an intermediate number of sheeting passes that maximises gluten development. There is thus scope for bakers to optimise the development of doughs containing bran, by adjusting bran particle size and sheeting, in order to minimise the detrimental effects of bran on bread quality.

It is surprising that the greatest expansion and springback are seen consistently after eight sheeting passes for all three roll gaps; one might expect that the change from a positive to a negative effect on development might depend on the roll gap. Clearly there may be subtle differences at the intermediate passes that were not examined; possibly at 6 mm, the maximum development occurs at roughly six or seven passes, and at nine or ten passes for a 12 mm gap. However, from the current study, there is no evidence that the optimum number of sheeting passes for maximum development is strongly affected by roll gap. This probably arises from the relatively consistent reduction ratio resulting from folding the sheeted dough between passes, such that the pattern of deformation is similar at the different roll gaps.

**Figure 7:**
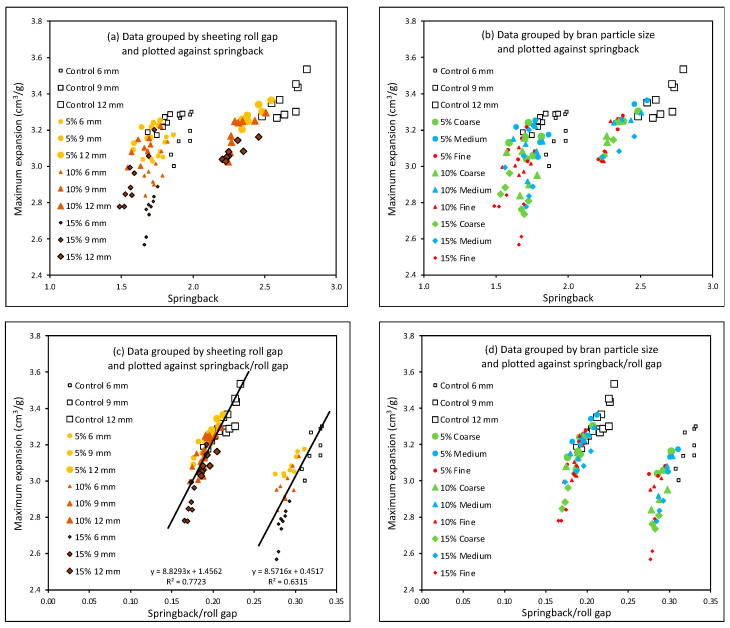
Maximum DDD expansion versus springback (**a**,**b**) and versus springback/roll gap (**c**,**d**), for doughs with different levels and particle sizes of bran, and sheeted at 6, 9 and 12 mm roll gaps. The data are grouped by sheeting roll gap (**a**,**c**) and by bran particle size (**b**,**d**).

Figure 7a shows maximum DDD expansion plotted against springback for the different roll gaps and bran levels, with bran particle sizes and the number of sheeting passes grouped together. The data do not show a single correlation but fall into three groupings that correspond to the three roll gaps, rather than the three bran particle levels or sizes (Figure 7b), with positive correlations between maximum expansion and springback within each group. This indicates that the effects of bran particle size and level on both springback and expansion are similar, but that the effects of sheeting roll gap on these two parameters is different.

Clearly, at each roll gap, the Control doughs without bran gave greater springback and expansion than doughs with bran. For the Control doughs and all three bran levels, the sheeting at 12 mm gave the greatest springback, although this did not translate into much greater expansion; relatively speaking, the increase in expansion was less than the increase in springback at 12 mm roll gap, compared with the smaller roll gaps. Sheeting at 9 mm gave similar levels of springback to sheeting at 6 mm, but greater expansion. 

Defining springback as dough thickness ex-sheeter divided by roll gap, for the purposes of defining a parameter that relates to gluten development, has a simple and intuitive appeal. However, it would be equally reasonable to suppose that roll gap may have a larger effect, such as a squared relationship, with gluten development. Figure 7c,d plots DDD expansion against springback/roll gap (in effect, against dough thickness/roll-gap^2^), which gives a dramatic regrouping. This alternative presentation has had the effect of bringing the data for roll gaps of 9 and 12 mm together, such that the data from different bran levels, particle sizes, roll gaps and number of sheeting passes all fall onto the same line. Meanwhile, the data for the 6 mm roll gap form an equally strong, but separate, correlation that similarly combines the effects of bran level, particle size and number of sheeting passes into a single relationship. Figure 7c shows the line of best fit, with very similar slopes for both groups and a higher R^2^ for the 9 and 12 mm data, explaining 77% of the variation arising from the four factors: bran level, bran particle size, roll gap and number of sheeting passes.

This separation of the 6 mm data from the 9 and 12 mm data may indicate that sheeting is behaving qualitatively differently at the smaller roll gap. It would be of interest to explore intermediate roll gaps to see exactly when this transition occurs, and hence to obtain a clearer understanding of the mechanisms by which sheeting enhances gluten development.

It is interesting to note that the qualitative difference in gluten development, as indicated by the different relationships between DDD expansion and springback, is related to roll gap rather than to some other factor. It might equally have been expected that such a clear qualitative difference would arise from the presence or absence of bran, or from a sudden transition occurring at a critical bran level, or from differences in bran particle size or (less probably) from exceeding a critical number of sheeting passes. However, all these effects are captured, for either the 6 mm roll gap or the 9 and 12 mm roll gaps together, in a single relationship, implying a single mechanism that is affected equivalently by bran level, bran particle size, number of sheeting passes and, in the case of 9 and 12 mm, roll gap, with a different manifestation of this single mechanism occurring at the 6 mm roll gap. 

More detailed modelling of sheeting, such as that presented by [8,9,10,26,30,31] may clarify a definition of springback that captures just the elastic component of the recoil phenomenon and is able to define a term that correlates with gluten development more unambiguously over all roll gaps; such modelling is beyond the scope of the current paper, but the work presented here gives data that would help test such a model. However, if the suggestion presented here is correct, that the gluten development is qualitatively different at the 6 mm roll gap, then the modelling of sheeting may fail to identify a mathematical description of springback that succeeds in reconciling the data from all the roll gaps.

### 3.3. Effects of Bran Particle Size, Sheeting Roll Gap and Number of Roll Passes on Baked Loaf Volume and Structure

**Figure 8:**
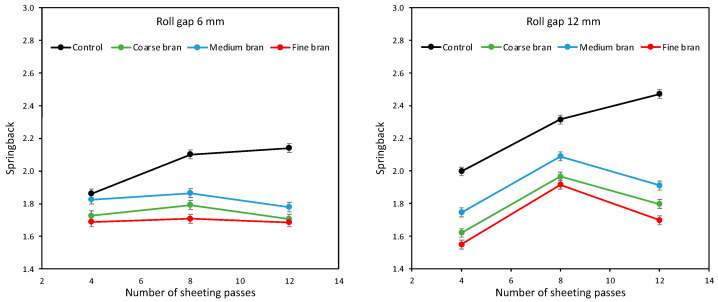
Average springback of doughs containing 10% of Coarse, Medium and Fine bran, sheeted at roll gaps of 6 mm (**left**) and 12 mm (**right**) for 4, 8 and 12 sheeting passes.

Doughs were prepared for baking trials with just 10% bran, and springback following sheeting was once again measured, as shown in Figure 8. As seen earlier in Figure 5, once again, sheeting was effective at developing the Control dough, while for doughs with bran, sheeting beyond 8 passes reduced springback, particularly for the 12 mm roll gap; and once again, the Medium bran gave the greatest springback and the Fine bran the least, for both roll gaps and all sheeting passes. The results are not identical to those of Figure 5, reflecting the inherent variability of dough studies, but they confirm the same broad trends.

**Figure 9:**
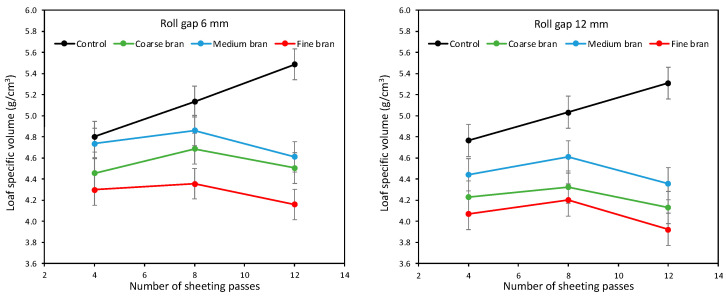
Average specific volume of baked loaves containing 10% Fine, Medium and Coarse bran, for doughs sheeted at roll gaps of 6 mm (**left**) and 12 mm (**right**) for 4, 8 and 12 sheeting passes.

**Figure 10:**
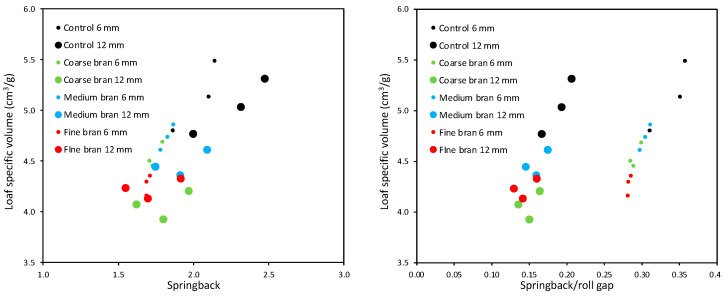
Average specific volume versus springback (**left**) and versus springback/roll gap (**right**), for doughs sheeted at roll gaps of 6 mm and 12 mm for 4, 8 and 12 sheeting passes.

Figure 9 shows the specific volume of baked loaves. Clearly the patterns closely mirror those from the DDD and springback results. This is confirmed by the correlations in Figure 10 (left), which show different correlations for the two roll gaps, supporting the point above that springback from different roll gaps cannot be directly compared and that the larger springback from sheeting at 12 mm did not translate into larger loaf volumes. Figure 10 (right) confirms that, as with DDD expansion, loaf specific volume is well correlated with springback/roll gap for each roll gap separately, such that springback/roll gap is a good indicator of the effects of bran particle size and number of sheeting passes on gluten development for a given roll gap, implying that the effects of these two parameters on gluten development are equivalent. However, the effect of sheeting on gluten development is qualitatively different under sheeting at a 6 mm roll gap compared with sheeting at a 12 mm roll gap.

**Figure 11:**
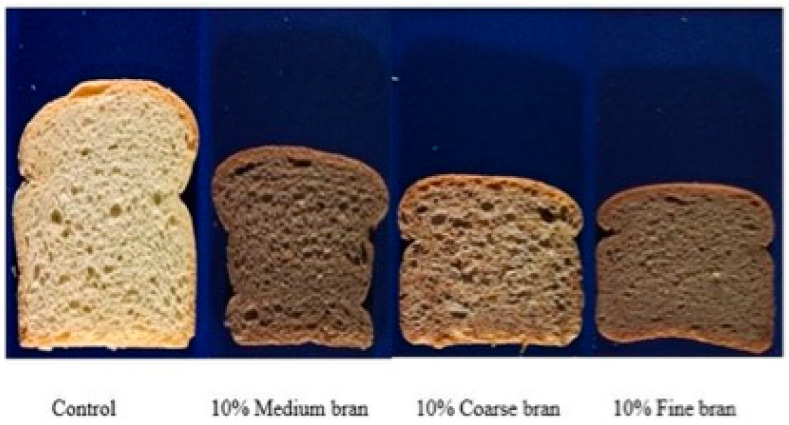
Effects of bran particle size on loaf volumes and crumb structures, for doughs containing 10% Coarse, Medium and Fine bran, sheeted at a roll gap of 12 mm for 8 sheeting passes.

Control doughs without bran gave the largest loaf volumes, and volume increased as sheeting increased from 4 to 8 to 12 passes at both roll gaps. Loaves were on average 5.2% larger after sheeting at a 6 mm roll gap compared with 12 mm, reflecting greater gluten development at the smaller gap. Bran decreased loaf volume, with Fine bran once again the most damaging and Medium bran the least, and with sheeting for 8 passes once again optimal compared with 4 or 12 passes. Figure 11 shows images of bread samples made without bran and with 10% Coarse, Medium and Fine bran, and with eight sheeting passes at a 6 mm roll gap. The images are ordered from highest to lowest volume, highlighting that Medium bran gave larger loaf volumes than Fine or Coarse, although still a lot lower than the Control without bran. Zhang and Moore [17] similarly found that their Medium bran gave the highest loaf volume and Fine bran the lowest.

**Figure 12:**
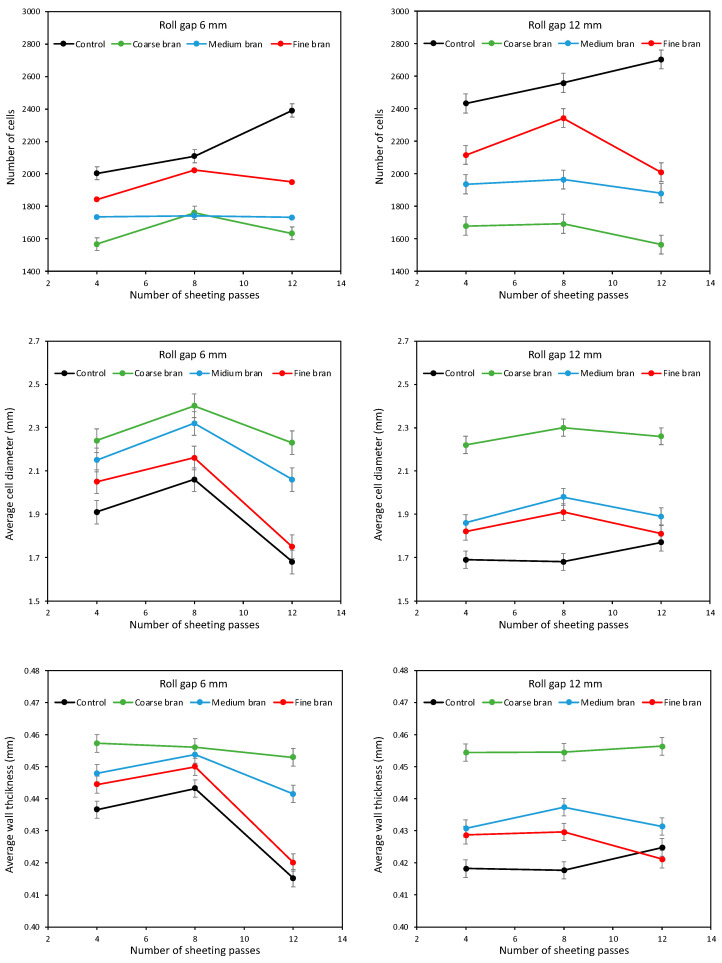
Average number of cells (**top**), average cell diameter (**middle**) and average wall thickness (**bottom**), for baked loaves containing 10% Coarse, Medium and Fine bran, sheeted at roll gaps of 6 mm (**left**) and 12 mm (**right**) for 4, 8 and 12 sheeting passes.

As well as giving a large loaf volume, good gluten development should retard coalescence of bubbles during proving and baking, leading to a large number of gas cells with small diameters and thin walls. Figure 12 shows the number of cells, the average cell diameter and the average wall thickness as affected by bran particle size and sheeting regime. For the Control dough, as the number of sheeting passes increased, the number of gas cells increased for doughs sheeted at both 6 and 12 mm roll gaps (Figure 12, top). This is in line with the increased loaf volumes reported in Figure 9 and reflects the enhanced gluten development that resists coalescence during proving and baking and retains large numbers of gas cells. It is also in agreement with Kilborn and Tipples [6] and Morgenstern et al. [9], who found sheeting gave finer crumb structures, the latter suggesting that sheeting also affects the bubble structure in the dough and that this may also influence the final gas cell structure in the baked loaf.

For the doughs with bran, gas cell numbers were lower than for the Control. In line with the results above for dough expansion, springback and loaf volume, sheeting for 8 passes gave more gas cells than for 4 or 12 passes. However, in this case it is clear that the Fine bran gave more gas cells than the Medium bran, with Coarse bran giving the lowest number of gas cells. Again, as well as reflecting the influence of the bran particles on gluten development, the effect of bran particle size on the initial bubble size distribution in the dough may translate into gas size distribution in the baked load. Bubble size distributions in doughs with bran have not been reported, but Campbell et al. [21] speculate that the presence of bran is likely to give larger bubbles in the dough.

The effects on average gas cell diameter and cell wall thickness are consistent between these two parameters; smaller gas cells correspond with thinner gas cell walls and a finer structure, while larger gas cells and thicker cell walls indicate a coarser structure. Broadly speaking, one would expect that better gluten development would result in more and smaller gas cells in the baked loaf, with thinner wall between the gas cells, i.e., a finer crumb structure. Following sheeting at both roll gaps, the Control bread had a higher number of gas cells than breads with bran, and a smaller average diameter and thinner cell walls, indicating a finer crumb structure as expected. When bran is added, the number of gas cells decreases and the average diameter and wall thickness increase, with all three parameters tending to show maxima at eight sheeting passes. In line with previous studies [16,17,18,19,34], Coarse bran gives the coarsest structure, with the fewest gas cells, largest average diameters and thickest walls, and Fine bran gives the finest structure—not forgetting, however, that bread quality is a combination of both loaf volume and crumb structure, and that Medium bran gave the largest loaf volume.

Small gas cell diameters are a positive advantage in bread loaves [35,36], and numerous studies recommend grinding bran to give finer crumb structures in wholemeal loaves [12,13,14,18,33,37,38], although Zhang and Moore [17] advise that an intermediate particle size may be better than bran that is as fine as possible. The current work supports the view that there may be an optimum bran particle size that maximizes loaf volume while maintaining an acceptable structure, and that there also appears to be an optimal sheeting regime that also maximises loaf volume and crumb fineness for bread formulations containing bran.

**Figure 13:**
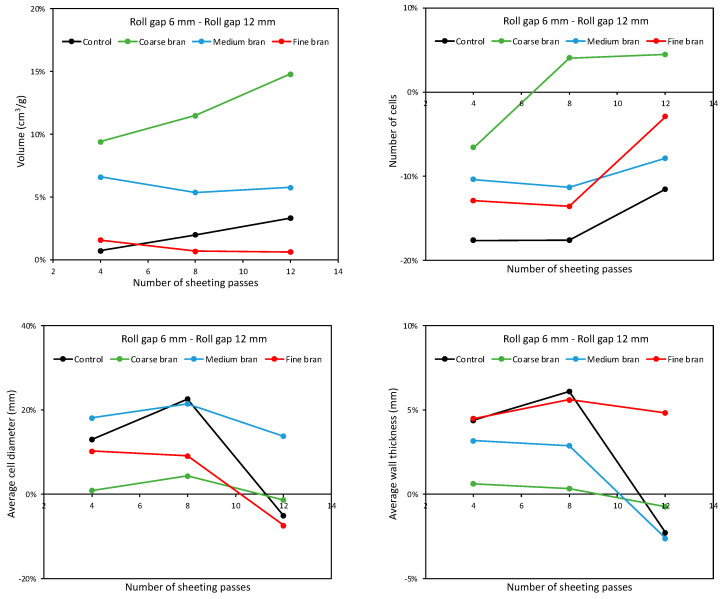
Percentage difference between sheeting at roll gaps of 6 and 12 mm, for loaf volume, average number of cells, average cell diameter and average wall thickness, for baked loaves containing 10% Fine, Medium and Coarse bran, sheeted for 4, 8 and 12 sheeting passes.

The sheeting regime comprises both the number of sheeting passes and the roll gap, both of which affect loaf volume and crumb structure. Figure 13 tries to clarify the effect of roll gap by plotting the percentage difference in loaf volume, number of gas cells, average gas cell diameter and average wall thickness when sheeting at 6 mm roll gap compared with 12 mm. Clearly, the 6 mm roll gap gives larger loaf volumes than the 12 mm gap, by on average 5.2%. For the Control dough, the increase in volume is small, increasing from 0.7% at 4 sheeting passes to 3.3% after 12 sheeting passes. For the dough with Fine bran, the increase is negligible, at around 1%. However, for doughs with Medium bran, the volume when sheeted at 6 mm is around 6% larger than when sheeted at 12 mm, and for Coarse bran, the volume increase is as much as 10–15%.

In the case of 8 and 12 sheeting passes, the increase appears to be because there are more gas cells when sheeting at 6 mm, compared with 12 mm, of much the same average diameter and wall thickness. By contrast, the Control dough, and the doughs with Fine or Medium bran, tend to have fewer and larger gas cells after sheeting at 6 mm. Thus, although the loaves are larger following sheeting at 6 mm, the crumb structure is less fine. The differences are, however, smaller after 12 sheeting passes.

The presence of bran, especially Fine bran, reduces the overall volume of loaves, and this decrease in the overall volume arises from smaller individual gas cells with a loaf, which produces a smaller and denser loaf. Although the presence of small cells in loaves is a beneficial feature, the decrease in overall volume is not. Bread quality is ideally reflected in a large loaf volume, combined with a fine crumb structure; good gluten development achieves both of these desirable features by resisting gas cell coalescence in order to retain gas in numerous small gas cells. However, the addition of bran disrupts this relationship, such that fine bran particles are particularly damaging to gluten and decrease gas retention overall, but the gas that is retained is dispersed into a larger number of small cells.

Sheeting is an operation available to bakers to incorporate into the breadmaking process in order to improve bread quality and the energy efficiency of the breadmaking process by enhancing gluten development. The current work has investigated for the first time the potential for the benefits of sheeting to be applied to wholemeal and high-fibre breads, in order to counteract the deleterious effects of fibre on gluten development and enhance the quality and palatability of these breads and their consumer acceptance, and hence to help deliver the benefits of high-fibre breads into the diet.

## 4. Conclusions

For doughs without bran, as the number of sheeting passes increased and the roll gap decreased, gluten development was enhanced, as evidenced from greater expansion in the DDD test, greater springback and larger baked loaves with finer crumb structures. This gives a clear indication of the effectiveness of sheeting on dough development, and a basis for quantifying development and optimising sheeting processes, thus maximising their benefits for bread quality and energy efficiency. 

For dough formulations with bran, maximum expansion and springback both increased from 4 to 8 sheeting passes, then decreased at 12 passes. Bran has a detrimental effect on dough expansion, with Fine bran particles more damaging than Coarse bran, and Medium the least damaging. However, sheeting can enhance the ability of bran-enriched doughs to expand, potentially offering a route to counteract the damaging effects of bran on gluten development, although over-development via sheeting becomes more of a risk with bran in the formulation. 

A single relationship between springback and DDD expansion was seen for doughs without bran and with bran at varying levels and particle sizes and sheeted for different numbers of passes and at 9 and 12 mm roll gaps, while the data for sheeting at 6 mm roll gap showed a different relationship that again incorporated the effects of bran level, particle size and number of sheeting passes. This implies that the effect on gluten development is equivalent from these different factors, but qualitatively different at 6 mm compared with the larger sheeting roll gaps.

The effects of bran on expansion capacity during proving were translated into effects on final baked loaf volume and structure, with Medium bran giving the largest volumes, while effects on the number and average diameter of gas cells and the average wall thickness showed complex interactions with volume. The results demonstrate how bakers can exploit and optimise sheeting to enhance bread quality, particularly for healthy high-fibre breads in which the presence of fibre damages gluten development, loaf quality and consumer acceptance. The practical implementation of sheeting in bakery production lines remains a challenge, but the approaches demonstrated here for optimising the benefits of sheeting, particularly for high-fibre breads, may help to strengthen the commercial case for sheeting and its successful implementation in the bakery.

## Figures and Tables

**Table 1:** Experimental plan for investigating effects of bran particle size (Coarse, Medium and Fine), level, roll gap and number of sheeting passes on dough development.

Day	Size of Bran	% Bran	Roll Gap	Number of Sheeting Passes	Number of Trials
1	Control + 3 particle sizes	5%	6	4, 8, 12	12
2	Control + 3 particle sizes	5%	9	4, 8, 12	12
3	Control + 3 particle sizes	5%	12	4, 8, 12	12
4	Control + 3 particle sizes	10%	6	4, 8, 12	12
5	Control + 3 particle sizes	10%	9	4, 8, 12	12
6	Control + 3 particle sizes	10%	12	4, 8, 12	12
7	Control + 3 particle sizes	15%	6	4, 8, 12	12
8	Control + 3 particle sizes	15%	9	4, 8, 12	12
9	Control + 3 particle sizes	15%	12	4, 8, 12	12

## Data Availability

Data are contained within the article.
